# p130Cas/Cyclooxygenase-2 axis in the control of mesenchymal plasticity of breast cancer cells

**DOI:** 10.1186/bcr3342

**Published:** 2012-10-26

**Authors:** Brigitte Bisaro, Maura Montani, Georgia Konstantinidou, Cristina Marchini, Lucia Pietrella, Manuela Iezzi, Mirco Galiè, Francesca Orso, Annalisa Camporeale, Shana M Colombo, Paola Di Stefano, Giusy Tornillo, Maria P Camacho-Leal, Emilia Turco, Daniela Taverna, Sara Cabodi, Augusto Amici, Paola Defilippi

**Affiliations:** 1 Department of Molecular Biotechnology and Health Sciences, University of Torino, Via Nizza 52, Torino, 10126, Italy; 2 Department of Bioscience and Biotechnology, University of Camerino, Via Gentile III da Varano, Camerino 62032, Italy; 3 Aging Research Centre, G. d'Annunzio University, Via dei Vestini 31, Chieti, 66013, Italy; 4 Dipartimento di Scienze Neurologiche, Neuropsicologiche, Morfologiche e Motorie, University of Verona, Piazzale L.A. Scuro 10, Verona 37134, Italy

## Abstract

**Introduction:**

Intrinsic plasticity of breast carcinoma cells allows them to undergo a transient and reversible conversion into mesenchymal cells to disseminate into distant organs, where they can re-differentiate to an epithelial-like status to form a cohesive secondary mass. The p130Cas scaffold protein is overexpressed in human ER+ and HER2+ breast cancer where it contributes to cancer progression, invasion and resistance to therapy. However, its role in regulating mesenchymal aggressive breast cancer cells remains to be determined. The aim of this study was to investigate the molecular and functional involvement of this adaptor protein in breast cancer cell plasticity.

**Methods:**

We used silencing strategies and rescue experiments to evaluate phenotypic and biochemical changes from mesenchymal to epithelial traits in breast tumor cell lines. In the mouse A17 cell model previously related to mesenchymal cancer stem cells and basal-like breast cancer, we biochemically dissected the signaling pathways involved and performed functional *in vivo *tumor growth ability assays. The significance of the signaling platform was assessed in a human setting through the use of specific inhibitors in aggressive MDA-MB-231 subpopulation LM2-4175 cells. To evaluate the clinical relevance of the results, we analyzed publicly available microarray data from the Netherlands Cancer Institute and from the Koo Foundation Sun Yat-Sen Cancer Center.

**Results:**

We show that p130Cas silencing induces loss of mesenchymal features, by downregulating Vimentin, Snail, Slug and Twist transcriptional factors, resulting in the acquirement of epithelial-like traits. Mechanistically, p130Cas controls Cyclooxygenase-2 transcriptional expression, which in turn contributes to p130Cas-dependent maintenance of mesenchymal phenotype. This cascade of events also compromises *in vivo *tumor growth through inhibition of cell signaling controlling cell cycle progression. c-Src and JNK kinases are sequential players in p130Cas/ Cyclooxygenase-2 axis and their pharmacological inhibition is sufficient to downregulate Cyclooxygenase-2 leading to an epithelial phenotype. Finally, *in silico *microarray data analysis indicates that p130Cas and Cyclooxygenase-2 concomitant overexpression predicts poor survival and high probability of breast tumor recurrence.

**Conclusions:**

Overall, these data identify a new p130Cas/Cyclooxygenase-2 axis as a crucial element in the control of breast tumor plasticity, opening new therapeutic strategies leading to inhibition of these pathways in aggressive breast carcinoma.

## Introduction

p130Cas is a tyrosine phosphorylated scaffold molecule originally identified in cells transformed by v-c-Src and v-Crk oncogenes [1-3]. p130Cas structural motifs and its posttranslational modifications enable interactions with many proteins leading to multi-protein complexes that in normal cells modulate cell motility, survival and proliferation [3]. In addition, p130Cas acts as a primary force sensor, transducing force into mechanical extension [4].

Extensive work on cancer cell models show that p130Cas is involved in cancer initiation, progression and metastasis formation [3]. p130Cas is necessary for transformation by several oncogenes, such as c-Src [5] and Her2 [6,7] as well as the oncogenic fusion protein nucleophosmin (NPM1)-anaplastic lymphoma receptor tyrosine kinase (ALK) [8]. Recently, p130Cas has been shown to be required for K-Ras, b-Raf, PTEN and PIK3CA oncogene-dependent proliferation [9]. Moreover, we have demonstrated that p130Cas is required for driving invasion and metastasis formation of HER2-transformed cells [10]. Finally, overexpression of p130Cas contributes to the development of human breast cancer [3]. It has been recently reported that in breast tumors overexpression of both Her2 and p130Cas is associated with increased proliferation, metastasis and poor prognosis [10,11]. Moreover, high levels of p130Cas have also been associated with resistance to the cytotoxic agent doxorubicin [12] and to anti-estrogen receptor (ER) therapy [13,14].

During metastasis dissemination, epithelial cancer cells can undergo a transient and reversible conversion into individual, motile and invasive mesenchymal cells to detach from the primary tumor, to disseminate into distant organs, and to form a cohesive secondary mass at a metastatic site, where they can re-differentiate to an epithelial-like status [15-19]. These processes, collectively defined as epithelial-mesenchymal (EMT) and mesenchymal-epithelia transition (MET), respectively, have been shown to be driven by coding and noncoding genes [20]; however, the regulatory program that controls tumor cell plasticity is not completely understood.

We previously established a carcinoma-derived mesenchymal tumor cell line, called A17, from a mammary carcinoma spontaneously developed in Balb-NeuT transgenic mice. These cells express cytokeratin 14 suggesting a myoepithelial origin, but not E-cadherin, indicating a partial transdifferentiation toward a mesenchymal phenotype [21]. The mesenchymal phenotype of A17 cells has been related to mesenchymal cancer stem cells and basal-like breast cancer [22,23]. Moreover, these cells significantly overexpress Cyclooxygenase-2 (Cox-2), a mesenchymal hallmark in tumors, whose relevance in growth, vasculogenesis and invasiveness has been widely documented in various types of carcinoma, both in clinical and experimental studies [24]. A human model of mesenchymal basal-like breast cancer is represented by the human lung metastatic MDA-MB-231 subpopulation LM2-4175 cells [25,26]. These cells also overexpress Cox-2 [25]. Here, we show that in these cells p130Cas silencing is sufficient to induce a switch from mesenchymal to epithelial features, to downregulate Cox-2 expression and mesenchymal markers and to impair *in vivo *tumor growth properties. Finally, we demonstrate that the concomitant expression of p130Cas and Cox-2 correlates with poor prognosis of human breast tumors. Taken together, these data describe a new role of p130Cas in EMT and cancer progression through the regulation of Cox-2 expression.

## Materials and methods

### Antibody and reagents

p130Cas mAbs have been previously described [6]. mAbs to Vinculin were from Millipore (Billerica, MA, USA). Abs to c-Src, p-Tyr PY99, Cyclin D1, Snail, Slug, Twist and Actin were from Santa Cruz Biotechnologies (Palo Alto, CA, USA). pTyr416 c-Src and pJnk (Thr183/Tyr185) Abs were from Cell Signaling (Beverly, MA, USA) and Abs to Cox-2 from Cayman Chemical (Ann Arbor, MI, USA). Secondary antibodies conjugated with peroxidase were from Sigma-Aldrich (St. Louis, MO, USA). Collagen I was from BD Trasduction Laboratories (Franklin Lakes, NY, USA). Doxycycline was purchased from Sigma-Aldrich.

### Cell cultures

A17 cells were cultured in DMEM-20% FCS and LM2-4175 in DMEM-10% FCS. Doxycycline at a concentration of 1 microgram/ml was directly added to medium and medium was changed every two to three days. The specific inhibitors of c-Src (SU6656) or JNK (SP600125) were used at a final concentration of 10 micromolar and 40 micromolar respectively for 16 hrs. Live images at 10X, 20X, magnification were collected with a Zeiss microscopy (Oberkochen, Germany).

### Generation of lentiviruses

Viral particles of pLVTHM carrying shRNA sequences (Ctr shRNA, p130Cas shRNA, or Cox-2 shRNA) were produced as described in [6]. For Cox-2 downregulation the sequences used were: GCTGTTCCAATCCATGTCAAA (COX-2 shRNA) and TCTCGCTTGGGCGAGAGTAAGCTC (Ctr shRNA).

For p130Cas and Cox-2 expression, human p130Cas cDNA, mouse p130Cas cDNA fused with GFP or human Cox-2 cDNA, respectively, were cloned into pCCL lentiviral vector, and viral particles production was performed as described above.

For silencing p130Cas in LM2-4175 cells, the human shRNA sequence (5'CCTTGCAGTACCCATCGCCTT3') was inserted into pLKO vector purchased from Open Biosystems (Thermo Fisher Scientific, Waltham, MA, USA). Lentiviruses were produced according to manufacturer's instructions.

### RNA isolation and qRT-PCR for mRNA detection

Total RNA was isolated from cells using TRIzol™ Reagent (Invitrogen Life Technologies, Carlsbad, CA, USA). 1 μg of DNAse-treated RNA (RQ1 RNase-Free DNase kit, Promega, Madison, WI, USA) was retrotranscribed with High Capacity cDNA Reverse Transcription Kit (Invitrogen Life Technologies, Carlsbad, CA, USA). Quantitative PCR was performed on an Applied Biosystems, 7900HT Fast Real-Time PCR System (standard settings) using the Universal Probe Library system (Roche Italia, Monza, Italy) and Platinum™ Quantitative PCR SuperMix-UDG (Invitrogen Life Technologies, Carlsbad, CA, USA). Results were analyzed with the 2^−ΔΔCt ^method using the 18S rRNA pre-developed TaqMan assay (Invitrogen Life Technologies, Carlsbad, CA, USA) as an internal control. The median expression across samples was used as calibrator. The following primers and probes were used: Cox-2, forward: GATGCTCTTCCGAGCTGTG reverse: GGATTGGAACAGCAAGGATTT, probe number 45; E-cadherin: forward: ATCCTCGCCCTGCTGATT and reverse: ACCACCGTTCTCCTCCGTA, probe number 18.

### Luciferase assay

To generate Cox-2 promoter luciferase reporter plasmids, two different Cox-2-promoter fragments were generated by PCR, using A17 genomic DNA as template, and the following primers: forward (-3195) 5'-CGCGCTCGAGTTTTATTGTTCTGCCCTCATGTGT-3'; forward (-965) 5'-CGCGCTCGAGCAACACAAACACAGTAGGAAGATA-3'; reverse (+39) 5'-CGCGAAGCTTGACTGACTCCTGAAGCTCTTAGCT-3'. The fragments (-3195 bp/-965 bp to +39 bp), respectively were cloned into pGL3-control vector expressing a firefly luciferase (Promega, Madison, WI, USA) using XhoI and HindIII restriction enzymes. The sequences of all constructs were confirmed by sequencing (BMR Genomics, Padova, Italy).

Luciferase activity was determined using a luciferase assay system (Promega, Madison, WI, USA) according to the manufacturer's protocol. Briefly, silenced cells seeded in 24-well plates (cells/well) were transiently transfected with Cox-2-promoter luciferase reporter plasmids with Lipofectamine 2000 (Invitrogen Life Technologies, Carlsbad, CA, USA). Upon 65 hrs of doxycycline treatment, luciferase assay was performed using the luciferase assay system (Promega, Madison, WI, USA) in a Berthold LB 953 luminometer. pGL3-control vector (Promega, Madison, WI, USA), in which the luciferase expression is driven by SV40 promoter, was used as positive control. Luciferase activity was expressed as relative light units per mg of cell proteins as determined by Bio-Rad Protein Assay Dye Reagent (Bio-Rad, Hercules, CA, USA). Each experiment was prepared in triplicate, and data are expressed as means ± SEM. Statistical significance was assessed using a Student's *t *test.

### Immunoblotting analysis

Protein extracts and western blots were performed as described in [6]. For tumor protein extraction, tissues were removed, frozen in liquid nitrogen, and homogenized in lysis buffer. Densitometric analysis was performed using the GS 250 Molecular Imager (Bio-Rad, Hercules, CA, USA)

### Cell adhesion assay *in vitro*

Adhesion assays were performed as described in [11] on dishes coated with 10 microgram/ml Collagen I.

### *In vivo *tumor growth

Fvb-Neu mice were challenged subcutaneously in the left inguinal region with 10^5 ^A17 Ctr shRNA, A17 p130Cas shRNA or A17 Cox-2 shRNA cells. The incidence and growth of tumors were evaluated twice weekly by measuring with calipers for the two perpendicular diameters. Mice water supplemented with doxycycline (0.1 mg/mL) was protected from light and changed every two to three days. The use of animals was in compliance with the Guide for the Care and Use of Laboratory Animals published by the US National Institutes of Health and was approved by the Animal Care and Use Committee of Camerino University

### Whole mount analysis, histology, and immunohistochemistry

Histology and immunohistochemistry preparations were performed as previously described [27].

For immunohistochemistry, these sections were incubated for 30 min with primary antibodies. After washing, they were overlaid with biotinylated goat anti-rat or anti-rabbit immunoglobulin (Vector Laboratories, Burlingame, CA, USA) for 30 min. Unbound antibodies were removed, and the slides were incubated with avidin-biotin complex/alkaline phosphatase (Dako, Milan, Italy).

### *In silico *analysis

Publicly available microarray data from the Netherlands Cancer Institute of 295 early-stage breast cancer biopsies (GSE2034) [28] and from the Koo Foundation Sun Yat-Sen Cancer Center (KFSYSCC) of 327 breast cancer tissues (GSE20685) [29] were used. Before analysis, the dataset was gene mean centered by subtracting the mean value for each gene across all samples of the compendium from all data points, so that in all cases expression values of each data point were reported as positive or negative depending on whether it was higher or lower than the mean value of that gene across the samples.

Statistical analysis was performed using a log-rank (Mantel-Cox) test.

### Statistical analysis

The results are representative of at least three independent experiments performed in triplicate and are expressed as the means ± SEM. Statistical analysis of the data was performed using a Student's *t *test.

## Results and discussion

### p130Cas silencing causes loss of mesenchymal features of breast cancer cells

**Figure 1:**
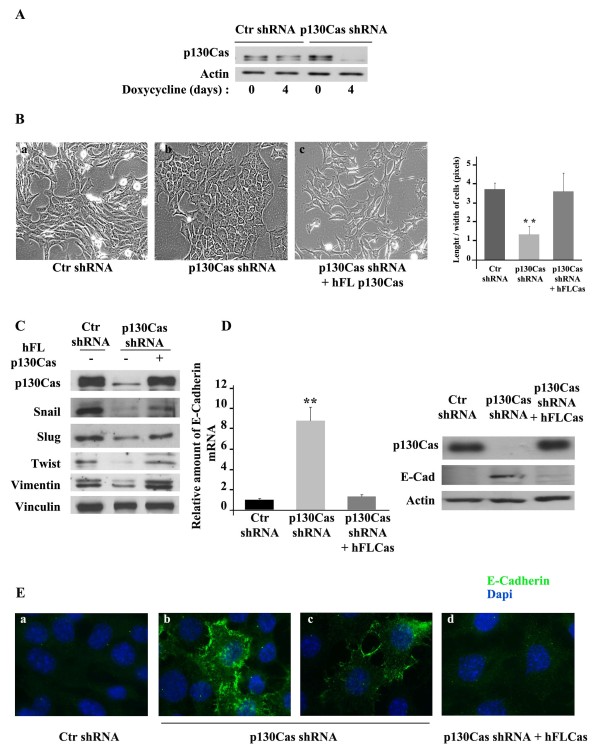
**p130Cas silencing promotes acquirement of epithelial traits in A17 cells**. **(A) **Extracts from A17 cells expressing scramble (Ctr shRNA) or p130Cas shRNAs (p130Cas shRNA) treated (days: 4) or not (days: 0) with doxycycline were blotted with antibodies to p130Cas. Actin was used as loading control. **(B) **Representative images of one out of ten experiments performed in A17 cells as in (A) **(a, b) **and p130Cas silenced cells reconstituted with human full-length (hFL) p130Cas (p130Cas shRNA + hFL p130Cas) **(c) **cultured in presence of doxycycline for four days (20X magnification). Right panel: Quantification of length/width ratio on five distinct microscope fields in each condition (**P value <0.001). **(C) **Extracts from cells as in (B) were probed with antibodies to p130Cas, Snail, Slug, Twist, Vimentin, and normalized with Vinculin. **(D) **Left panels: quantification of E-cadherin mRNA by qRT-PCR in Control shRNA, p130Cas shRNA and in p130Cas silenced cells reconstituted with human full-length p130Cas (hFLCas) A17 cells (**P <0.001). Right panel: cell extracts were blotted with antibodies to p130Cas, and E-cadherin. Actin was used as loading control. **(E) **Representative images of E-cadherin immunofluorescence performed on cells as in (D) (100X magnification).

To investigate the role of p130Cas in mesenchymal breast cancer cells, we generated cells expressing doxycycline-inducible control or p130Cas shRNA sequences [6], resulting in p130Cas silencing of about 90% (Figure 1A). Remarkably, upon four days of doxycycline treatment, p130Cas silenced cells underwent a switch from an elongated mesenchymal phenotype to a polygonal epithelial-like shape (Figure 1B, panels a, b) that reverted upon re-expression of p130Cas in silenced cells (Figure 1B, panel c), indicating that p130Cas tuning can control mesenchymal breast cancer cell plasticity.

p130Cas silenced cells revealed decreased expression of the transcriptional factors Snail, Slug and Twist, and of the mesenchymal marker Vimentin, whose levels were restored by re-expression of p130Cas (Figure 1C), or by washing out doxycycline from A17 culture medium (Figure S1 in Additional file 1). Snail, Slug and Twist are known to repress E-cadherin expression during EMT [20,30]. Quantitative real-time PCR experiments and western blot analysis (Figure 1D) showed that E-cadherin was induced both at mRNA and protein levels upon p130Cas silencing. Consistently, when p130Cas was re-expressed in silenced A17 cells, E-cadherin expression was strongly downregulated, returning to control levels. Immunofluorescence staining clearly showed that upon p130Cas silencing E-cadherin expression becomes detectable in A17 cells with a strong plasma membrane staining (Figure 1E, panels b and c) that is totally missing in control (panel a) and in p130Cas reconstituted cells (panel d). Thus p130Cas can modulate expression of mesenchymal/epithelial markers, resulting in a reversible transition from mesenchymal to epithelial features.

p130Cas has been already shown to play a role in the intrinsic plasticity that allows cells to switch from epithelial to mesenchymal phenotype in pancreatic cancer cells [31], while the second member of the Cas protein family NEDD9 controls EMT in breast [32,33], and melanoma [34] cancer cells. Remarkably, by mass spectrometry-based profiling, p130Cas tyrosine phosphorylation has been described to be elevated in basal breast cancer cells [35]. Genome-wide transcriptional profiling of a large set of human breast cancer cell lines confirms that EMT features are mostly associated with basal-like tumors [36,37], suggesting a link between p130Cas expression and basal breast tumors.

### p130Cas-dependent Cox-2 expression is involved in maintenance of mesenchymal phenotype

**Figure 2:**
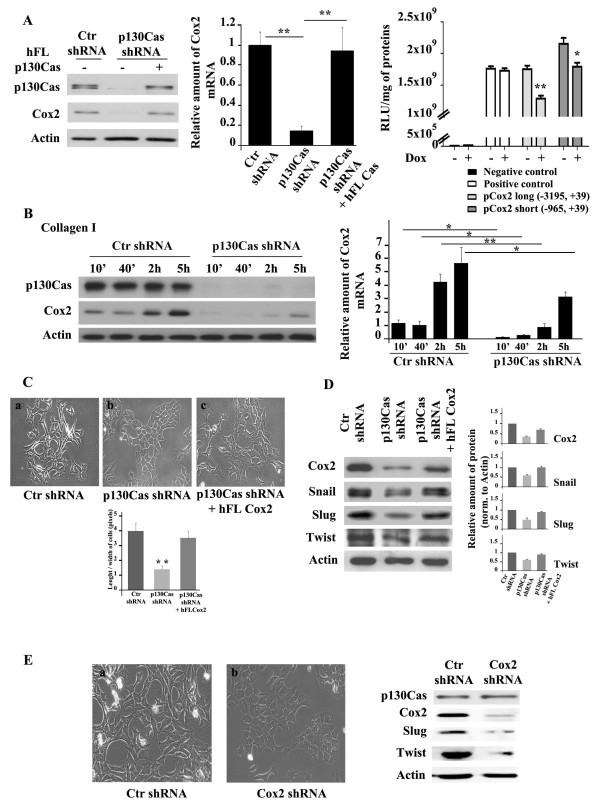
**p130Cas-dependent Cox-2 transcriptional expression sustains mesenchymal features of A17 cells**. **(A) **Left panels: extracts from A17 cells expressing scramble (Ctr shRNA), p130Cas shRNAs (p130Cas shRNA) or reconstituted with human full-length (hFL) p130Cas (p130Cas shRNA + hFL p130Cas) were blotted with antibodies to p130Cas and Cyclooxygenase-2 (Cox-2). Actin was used as loading control. Middle panel: quantification of Cox-2 mRNA by qRT-PCR in cells as in (A) (**P <0.001). Right panel: luciferase activity assay in p130Cas silenced cells transfected with pGL3 vectors carrying luciferase reporter gene downstream of a short (-965, +39) or long (-3195, +39) stretch of Cox-2 promoter in presence or absence of doxycycline (*P <0.05 and **P <0.001). Negative control: pGL3 vector lacking promoter. Positive control: pGL3 vector, in which the luciferase expression is driven by SV40 promoter. **(B) **Left panels: Ctr and p130Cas silenced A17 cells treated with doxycycline for four days were plated on Collagen I-coated dishes for different times. Cell extracts were analyzed by blotting with antibodies to Cox-2. Actin was used as loading control. Right panel: quantification of adhesion-dependent Cox-2 mRNA by qRT-PCR (*P <0.05 and **P <0.001). **(C) **Upper panels: representative images of one out of three experiments performed in Ctr shRNA cells **(a)**, p130Cas shRNA cells **(b) **or p130Cas shRNA cells reconstituted with hFL Cox-2 (p130Cas shRNA + hFL Cox2) **(c) **cultured in presence of doxycycline for four days (20X magnification). Lower panel: quantification of length/width ratio on five distinct microscope fields in each condition (**P <0.001). **(D) **Right panels: cell extracts were probed with antibodies to Cox-2, Snail, Slug, and Twist. Actin was used as loading control. Left panels: quantification analysis and statistic were performed on three independent experiments. **(E) **Left panels: representative images from A17 cells expressing scramble (Ctr shRNA) **(a) **or Cox-2 shRNAs (Cox-2 shRNA) **(b) **treated with doxycycline for four days (20X magnification). Right panels: extracts from Ctr or Cox-2 silenced A17 cells were blotted with antibodies to Cox-2, Slug, Twist, and Actin, used as loading control.

Cox-2 is frequently associated with aggressive breast cancer [38]. Cox-2 was found significantly overexpressed in A17 cells, where it correlates with their mesenchymal signature [22,23]. Interestingly, in p130Cas silenced cells the expression of Cox-2 markedly decreased, and was restored by re-expressing p130Cas (Figure 2A, left panel). qRT-PCR showed that in p130Cas silenced cells Cox-2 mRNA was reduced by 80% compared to control cells, and restored to control levels after p130Cas re-expression in silenced cells (Figure 2A, middle panel), suggesting that p130Cas exerts a transcriptional control on Cox-2 expression. Luciferase assays on two DNA fragments corresponding to a short (-965, +39) and a long (-3195, +39) Cox-2 promoter indicated that p130Cas silencing significantly decreased Cox-2 promoter activity (Figure 2A, right panel). Adhesion-dependent Cox-2 induction has been previously described [39-41]. Consistently, plating control and p130Cas silenced cells on Collagen I-coated dishes for different times, showed that Cox-2 induction both at mRNA and protein levels and was markedly delayed and decreased in p130Cas silenced cells (Figure 2B). Taken together, these results show that p130Cas is a key upstream element in the regulation of Cox-2 expression in breast cancer cells. As Cox-2 has been proposed as a mediator of breast tumor epithelial-stroma interactions, which promote growth and progression of *in situ *tumors [42], these results suggest that p130Cas can behave as a master regulator of tumor/microenvironment interactions.

Interestingly, the p130Cas-dependent expression of Cox-2 is instrumental for the regulation of breast cancer cells plasticity. Indeed, re-expression of Cox-2 in p130Cas silenced cells reverted cells to a mesenchymal morphology (Figure 2C, panel c) and restored Snail, Slug and Twist expression (Figure 2D). Accordingly, cells expressing doxycycline-inducible Cox-2 shRNAs in which Cox-2 was knocked down by about 90% (Figure 2E, right panels), exhibited a clear switch from an elongated to a polygonal epithelial shape (Figure 2E, panel b). Moreover, these cells showed marked downregulation of Slug and Twist transcriptional factors, while p130Cas expression was not affected (Figure 2E, right panels). These results indicate that p130Cas controls Cox-2 expression and that Cox-2 is involved in p130Cas-dependent maintenance of mesenchymal phenotype, thus establishing a p130Cas/Cox-2 axis that sustains the mesenchymal features of breast cancer cells.

### The p130Cas/Cox-2 axis controls *in vivo *tumor properties of breast cancer cells

**Figure 3:**
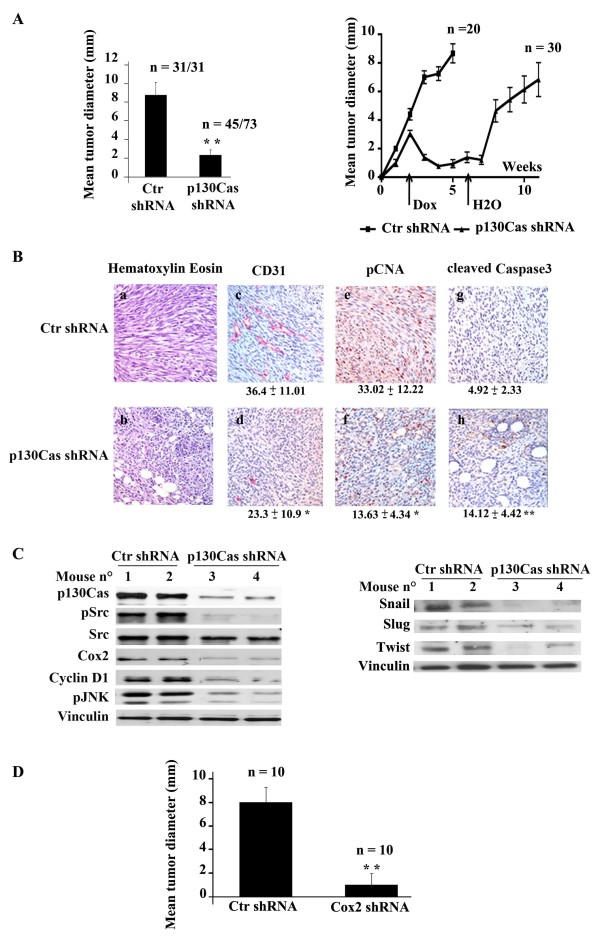
**p130Cas/Cyclooxygenase-2 (Cox-2) modulates A17 *in vivo *growth**. **(A) **Left panel: A17 cells expressing Ctr shRNA or p130Cas shRNAs were orthotopically injected in FVB-NeuN mice. Doxycycline has been supplied in drinking water at the time of injection. Tumor mean diameter at 21 days after injection is reported on the y-axis (**P <0.001). Right panel: cells were orthotopically injected in mice and allowed to grow for two weeks giving rise to tumors with a mean diameter of 1 mm. Doxycycline was then added to water of mice to induce the expression of Ctr or p130Cas shRNA and tumors diameter were measured every week for four weeks. Finally, at the beginning of the sixth week, doxycycline was removed and tumor growth was measured for other six weeks. n/n: number of measured tumors/injected mice for each group. **(B) **Representative images of hematoxylin and eosin staining and immunohystochemical analysis of Ctr or p130Cas silenced tumor sections. The antibodies against CD31 **(c, d)**, pCNA **(e, f) **and cleaved caspase-3 **(g, h) **were used on paraffin-embedded sections (4X magnification). Quantification of immunohystochemical analysis was performed by counting CD31 positive vessels in eight different fields at 200X magnification, by expressing pCNA percentage of positive nuclei on the total nuclei, and by counting cleaved caspase-3-positive cells of 20 fields at 400X magnification. **(C) **Protein extracts from Ctr or p130Cas silenced tumors were blotted with antibodies to p130Cas, pSrc (pTyr416), c-Src, Cox-2, Cyclin D1, pJnk (Thr183/Tyr185), Snail, Slug, Twist, and Vinculin as loading control. **(D) **A17 cells expressing Ctr shRNA or Cox-2 shRNAs were orthotopically injected in FVB-NeuN mice. Doxycycline had been supplied in drinking water at the time of injection. Tumor mean diameter at four weeks after injection is reported on the y-axis (**P <0.001). n: number of measured tumors for each group.

To investigate the role of p130Cas/Cox-2 axis on tumor growth, syngeneic mice were subcutaneously injected with 10^5 ^control or p130Cas silenced cells and treated with doxycycline in drinking water. Within three weeks, all the 31 mice injected with control cells gave rise to tumors with a mean diameter of 8 mm. In contrast, 38% (28 out of 73) of mice injected with p130Cas silenced cells did not give rise to detectable tumors and the remaining 45 mice developed small tumors, with a mean diameter of 2 mm (Figure 3A, left panel). Interestingly, p130Cas silencing was sufficient to halt tumor growth in mice that have already developed tumors with a diameter of 3 to 4 mm. Indeed, by adding doxycycline to drinking water two weeks after cell injection, p130Cas silenced tumors regressed, becoming undetectable by palpation within two to three weeks, while control tumors continued to grow (Figure 3A, right panels). Consistently, after doxycycline withdrawal p130Cas silenced tumors resumed growing. These data strengthen the *in vivo *relevance of p130Cas as a major regulator of the tumorigenic properties of mesenchymal breast cancer cells. We have previously shown that intranipple injection of p130Cas siRNAs in the mammary gland of Balb/c-NeuT mice significantly decreases the number of cancer lesions compared to glands injected with control siRNAs [6], with a significant downregulation of proliferative and survival pathways. Overall these data indicate that tight modulation of p130Cas levels can affect *in vivo *tumor properties of distinct breast cancer subtypes, implying the compelling need of studying its transcriptional regulation in normal mammary epithelial cells and in tumors in the near future.

Hematoxylin and eosin staining of tumor sections showed that tumors derived from p130Cas silenced cells consisted of cells with an epithelial-like shape, while the control tumors presented elongated, mesenchymal cells (Figure 3B, panels a, b). Moreover, immunohistochemistry analysis indicated that tumors from p130Cas silenced cells were characterized by decreased vascularization and proliferation (CD31 and pCNA staining), and increased apoptosis (*de novo *expression of Caspase-3) (Figure 3B, panels c-h).

Western blot analysis of p130Cas silenced tumors showed a significant *in vivo *p130Cas silencing together with Cox-2 downregulation, compromised activation of c-Src and JNK kinases and decreased expression of Cyclin D1 (Figure 3C, left panels). A parallel downregulation of Snail, Slug and Twist expression was also detected (Figure 3C, right panels), indicating that p130Cas silencing compromises tumor growth through inhibition of cell signaling controlling cell cycle progression and the acquirement of epithelial-like features. In parallel, syngeneic mice were subcutaneously injected with 10^5 ^Cox-2 silenced or control A17 cells and treated with doxycycline in drinking water. As shown in Figure 3D, while mice injected with control cells gave rise to tumors with a mean diameter of 10 mm within six weeks, mice injected with Cox-2 silenced cells give rise to barely detectable tumors. Taken together these data show that p130Cas/Cox2 axis controls *in vivo *survival and proliferative pathways of mesenchymal breast cancer cells and silencing of either p130Cas or Cox-2 is sufficient for switching cells to an epithelial state leading to impaired tumor growth.

### The p130Cas/Cox2 axis requires c-Src and JNK activities to sustain mesenchymal traits

**Figure 4:**
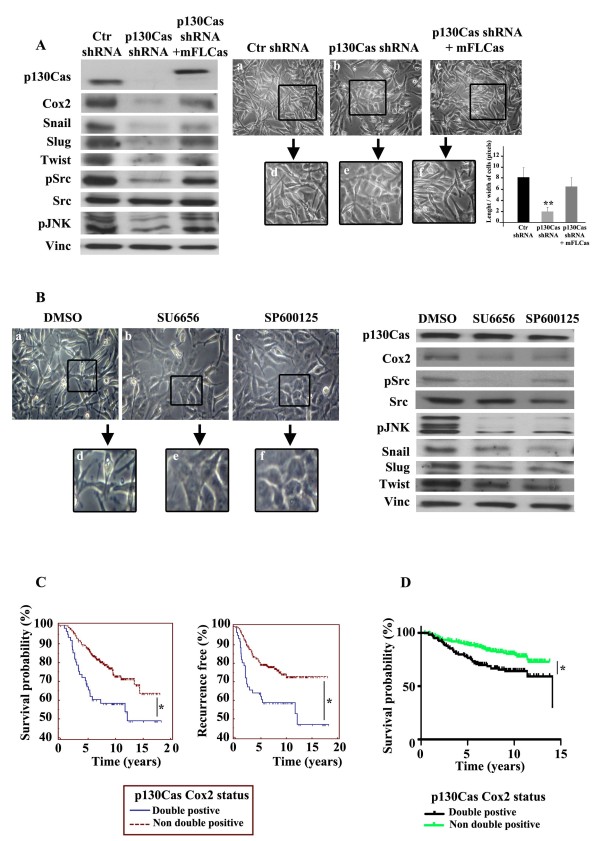
**p130Cas/Cyclooxygenase-2 (Cox-2) axis sustains mesenchymal traits and predicts poor outcome in human breast cancers**. **(A) **Left panels: extracts from LM2-4175 cells expressing scramble (Ctr shRNA), p130Cas shRNAs (p130Cas shRNA) or silenced p130Cas cells reconstituted with mouse full-length GFP. p130Cas (mFLCas) were blotted with antibodies to p130Cas, Cox-2, Snail, Slug, Twist, pSrc (pTyr416), pJnk (Thr183/Tyr185), and Vinculin as loading control. Right panels: representative images (10X magnification) of Ctr **(a)**, p130Cas silenced **(b)**, and p130Cas reconstituted **(c) **LM2-4175 cells. A 20X magnification of the squared field is shown **(d, e, and f)**. Quantification of length/width ratio on five distinct microscope fields in each condition (**P <0.001) is shown. **(B) **Left panels: representative images of LM2-4175 cells treated with DMSO **(a)**, 10 micromolar c-Src inhibitor (SU6656) **(b)**, or 40 micromolar JNK inhibitor (SP600125) **(c) **for 16 h. 20X magnification of the squared field are shown below **(d-f)**. Right panels: protein extracts were blotted with antibodies against p130Cas, Cox-2, pSrc (pTyr416), c-Src, pJnk (Thr183/Tyr185), Snail, Slug, Twist, and Vinculin as loading control. **(C) ***In silico *analysis of NKI dataset. Before analysis, the dataset was gene mean centered by subtracting the mean value for each gene across all samples of the compendium from all data points, so that in all cases expression values of each data point were reported as positive or negative depending on whether it was higher or lower than the mean value of that gene across the samples. Kaplan-Meier curves indicating survival probability of patients with tumors overexpressing p130Cas and Cox-2 (double positive), compared to tumors with an expression of p130Cas and Cox-2 lower than the mean value previously defined (non-double positive). **(D) ***In silico *analysis as in (C) on the Koo Foundation Sun Yat-Sen Cancer Center dataset.

To assess whether the p130Cas/Cox-2 axis is effective also in the human setting, we chose the human lung metastatic MDA-MB-231 subpopulation LM2-4175 [25,26] as they recapitulate A17 cell features with high levels of Cox-2 expression and a mesenchymal phenotype. Upon infection with lentiviral particles carrying human p130Cas shRNA, the marked downregulation of p130Cas was associated with a concomitant decrease in Cox-2, Snail, Slug and Twist (Figure 4A, left panels). Accordingly, p130Cas silenced cells reorganized in colonies that lost their elongated protrusions, acquiring a more polygonal shape (Figure 4A, panels b and e), as quantified by a marked decreased in length/width ratio. Re-expression of a mouse full-length p130Cas-GFP fused protein (mFLCas) in LM2-4175 p130Cas silenced cells, re-established Cox-2 and mesenchymal markers expression at the same level of control cells (Figure 4A, left panels), and consistently p130Cas reconstituted cells reacquired elongated protrusions (Figure 4A, panels c and f). Moreover, p130Cas silencing led to a strong reduction of c-Src and JNK activities (Figure 4A, left panels), similar to those observed in *in vivo *tumor grafts derived from p130Cas silenced A17 cells. Interestingly, cell treatment with specific inhibitors of c-Src (SU6656) or JNK (SP600125) activities for 16 hrs, caused a switch to an epithelial morphology similar to that observed upon p130Cas downregulation (Figure 4B, left panels). Consistent with the fact that Src and JNK controls Cox-2 expression (for review, [43]), both inhibitors caused downregulation of Cox-2, and a reduction in Snail, Slug and Twist expression (Figure 4B, right panels), without grossly affecting p130Cas levels. In addition, cells treated with the c-Src inhibitor SU6656 showed a decrease in JNK activity, while the JNK inhibitor SP600125 did not affect c-Src phosphorylation, suggesting that Src activity is upstream to JNK activation. Moreover, in A17 cells, luciferase assays revealed that the reporter expression driven by Cox-2 promoter was decreased by the use of Src inhibitor and practically abrogated with JNK inhibitor (Figure S2 in Additional file 1). Overall these data show that the p130Cas/Cox-2 axis is effective both in the mouse and in the human setting. c-Src and JNK kinases appear as sequential players in this axis and their pharmacological inhibition was sufficient to downregulate Cox-2 and to induce an epithelial phenotype. These results also suggest the potential clinical application of targeting c-Src through pharmacological inhibitors in breast tumors expressing high levels of p130Cas and Cox-2, the same strategy already proposed in HER2-positive trastuzumab-resistant tumors to overcome trastuzumab resistance [44].

Finally, in order to evaluate whether the p130Cas/Cox-2 axis has clinical relevance in human breast cancer, publicly available microarray data from the Netherlands Cancer Institute of 295 early-stage breast cancer biopsies [28] and from the Koo Foundation Sun Yat-Sen Cancer Center (KFSYSCC) of 327 breast cancer tissues [29] were analyzed. Kaplan-Meier curves showed that p130Cas and Cox-2 double positivity was associated with the lowest time survival (log-rank Van de Vijver *P *= 0.0025 and Kao *P *= 0.0071) (Figure 4C, D), and the highest frequency of recurrence (log-rank *P *= 0.0013) (Figure 4C), indicating that high levels of p130Cas/Cox-2 co-expression relates to the worst prognosis in breast cancer. Previous data have already shown that high levels of p130Cas correlate with intrinsic resistance to tamoxifen treatment in a large subset of estrogen receptor (ER)-positive human breast tumors (reviewed in [3]). Moreover, in human breast cancers overexpression of both HER2 and p130Cas is associated with poor prognosis [10].

## Conclusions

**Figure 5:**
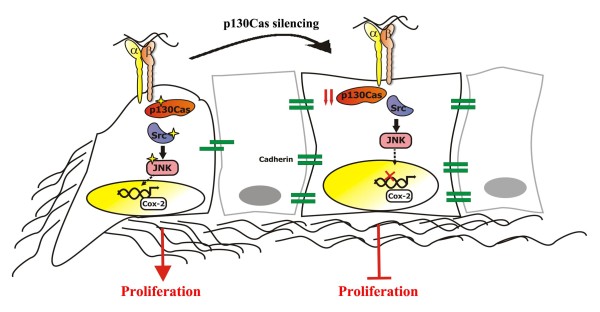
**Schematic model illustrating the role of p130Cas in sustaining the mesenchymal features of breast cancer cells**. p130Cas supports mesenchymal traits of breast cancer cells by sustaining Src, JNK activities (see stars), and expression of Cyclooxygenase-2 **(**Cox-2), leading to tumor growth. In contrast, low levels of p130Cas, preventing the activation of downstream signaling pathways, allow cells to acquire epithelial features, impairing tumor growth.

Overall in this work we demonstrate the involvement of p130Cas in mesenchymal breast cancer cell plasticity, highlighting a new pathway linking p130Cas to Cox-2 through c-Src and JNK activities (Figure 5). p130Cas is thus emerging as a critical player for onset and progression of many aggressive cancers, strengthening its relevance as an unfavorable prognostic marker and a putative therapeutic target, mostly in combination with high levels of ER, HER2 or Cox-2, respectively.

## Abbreviations

Cox-2: Cyclooxygenase-2; DMEM: Dulbecco's modified Eagle's medium; EMT: epithelial-mesenchymal transition; ER: estrogen receptor; FCS: fetal calf serum; GFP: green fluorescent protein; mAb: monoclonal antibody; MET: mesenchymal epithelial transition; qRT-PCR: quantitative real-time polymerase chain reaction; shRNA: short hairpin RNA; siRNA: small interfering RNA.

## Competing interests

The authors declare that they have no conflict of interest.

## Authors' contributions

BB design of the experiments, generation of lentiviruses and of silenced cells, re-expression experiments and draft manuscript writing; MM *in vivo *experiments with p130Cas silenced cells; GK generation of Cox-2 shRNA lentiviral constructs; CM characterization of A17 cells, luciferase assays for Cox-2 promoter; LP generation of Cox-2 shRNA lentiviral constructs; MI pathology analysis and IHC of tumor sections; MG *in silico *analysis of human dataset; FO *in vivo *experiments with p130Cas silenced cells; AC cell cycle analysis of silenced cells; SC cell culture and western blot analysis; PD design of the experiments and discussion; GT generation of Cox-2 silenced cells; MPCL discussion; ET generation of p130Cas shRNA and overexpressing lentiviral constructs and draft manuscript writing; DT design of the experiments and discussion; SC design of the experiments and discussion; AA *in vivo *experiments with p130Cas and Cox-2 silenced cells; PD design of the experiments, direction and manuscript writing. All authors read and approved the final manuscript.

## Supplementary Material

Additional file 1 **Supplementary Figures. Figure S1. p130Cas expression controls mesenchymal/epithelial behavior of A17 cells**. Morphological shape and biochemical analysis of A17 cells expressing scramble or p130Cas shRNAs upon doxycycline treatment or doxycycline wash-out. **Figure S2. c-Src and JNK regulate the reporter expression driven by Cyclooxygenase-2 (Cox-2) promoter**. Luciferase activity assay in A17 cells upon pharmacological treatments with Src or JNK inhibitors. Click here for file
