# Supplementary material for: p130Cas/Cyclooxygenase-2 axis in the control of mesenchymal plasticity of breast cancer cells
Source: Breast Cancer Res. 2012 Oct 26;14(5):R137. doi: 10.1186/bcr3342 (PMC4053116; doi:10.1186/bcr3342)
Supplement: Additional file 1 — Supplementary Figures. Figure S1. p130Cas expression controls mesenchymal/epithelial behavior of A17 cells. Morphological shape and biochemical analysis of A17 cells expressing scramble or p130Cas shRNAs upon doxycycline treatment or doxycycline wash-out. Figure S2. c-Src and JNK regulate the reporter expression driven by Cyclooxygenase-2 (Cox-2) promoter. Luciferase activity assay in A17 cells upon pharmacological treatments with Src or JNK inhibitors. [file bcr3342-S1.DOC]

**Supplementary Figures**


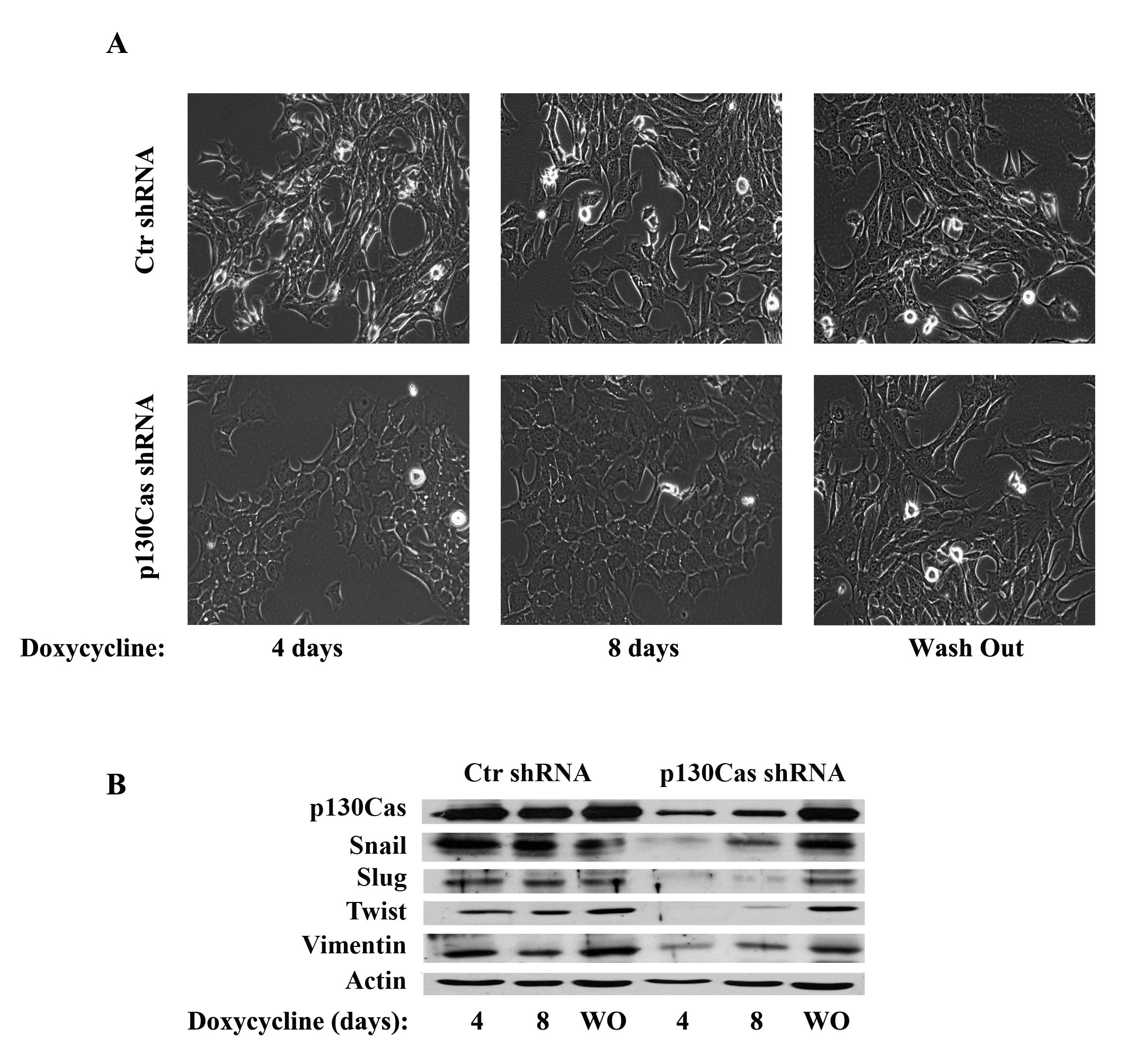


**S1**

**p130Cas expression controls mesenchymal/epithelial behavior of A17 cells.**

(A): Morphological shape of A17 infected cells expressing scramble (Ctr shRNA) or p130Cas shRNAs (p130Cas shRNA). Cells were treated with doxycycline for 4 days and then cultured for another 4 days in presence (8 days) or absence (Wash out) of doxycycline (20X magnification).

(B): Extracts from Ctr or p130Cas shRNA cells treated with doxycycline as in A were probed with antibodies to p130Cas, Snail, Slug, Twist, Vimentin, and normalized with Actin (WO: Wash out).


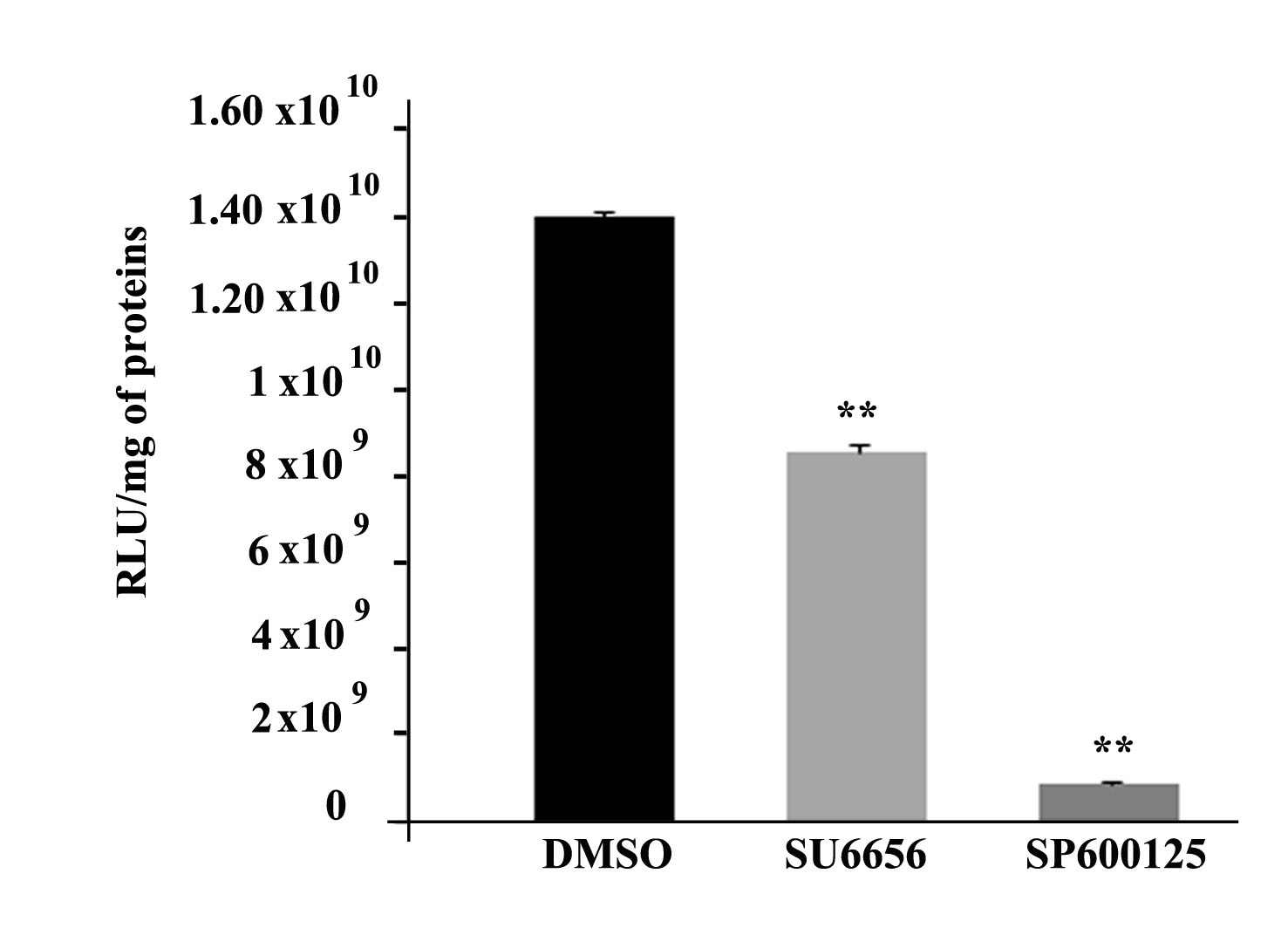


**S2**

**c-Src and JNK regulate the reporter expression driven by COX2 promoter.**

Luciferase activity assay in A17 cells, transfected with a pGL3 vector carrying luciferase reporter gene downstream of a long (-3195, +39) stretch of Cox-2 promoter, in presence of DMSO (control) or pharmacological treatments with 10mM of Src inhibitor (SU6656) and 40mM of JNK inhibitors (SP600125) for 20 hours (** p< 0.001).
